# In-capillary sample processing coupled to label-free capillary electrophoresis-mass spectrometry to decipher the native N-glycome of single mammalian cells and ng-level blood isolates

**DOI:** 10.21203/rs.3.rs-3500983/v1

**Published:** 2023-11-14

**Authors:** Alexander Ivanov, Anne-Lise MARIE, Yunfan Gao

**Affiliations:** Northeastern University

## Abstract

The development of reliable single-cell dispensers and substantial sensitivity improvement in mass spectrometry made proteomic profiling of individual cells achievable. Yet, there are no established methods for single-cell glycome analysis due to the inability to amplify glycans and sample losses associated with sample processing and glycan labeling. In this work, we developed an integrated platform coupling online in-capillary sample processing with high-sensitivity label-free capillary electrophoresis-mass spectrometry for N-glycan profiling of single mammalian cells. Direct and unbiased characterization and quantification of single-cell surface N-glycomes were demonstrated for HeLa and U87 cells, with the detection of up to 100 N-glycans per single cell. Interestingly, N-glycome alterations were unequivocally detected at the single-cell level in HeLa and U87 cells stimulated with lipopolysaccharide. The developed workflow was also applied to the profiling of ng-level amounts of blood-derived protein, extracellular vesicle, and total plasma isolates, resulting in over 170, 220, and 370 quantitated N-glycans, respectively.

## INTRODUCTION

Glycans are assemblies of linear and branched monosaccharide chains that govern molecular interactions and, therefore, cell communication, signal transduction, pathogen recognition, and immune responses ^1–3^. In living mammalian cells, glycosylation (catalyzed by glycosyltransferases and glycosidases) produces a highly complex and vast repertoire of cellular glycans, with a colossal structural diversity ^1^. Mammalian cells are covered with a dense layer of glycans and the proteins and lipids they are attached to, termed the *glycocalyx*, which is involved in various vital cellular processes ^4–6^. The type, size, structure, and charge of cell surface glycans may affect the biological properties of the cells and their susceptibility to potential viral infections ^2,4,7^. Mammalian glycoconjugates, located on the cell membrane and extra- or intracellular space, play crucial roles in physiological and pathological events ^2,4,5,7,8^. Alterations in the glycomic profiles of glycoproteins, e.g., overexpression of sialylated or core fucosylated glycans, or increased levels of complex-type branched glycans, may promote the acquisition of cellular features required for the malignant transformation of cells ^1,9–12^. Recent studies also reported altered glycosylation patterns in patients with Alzheimer’s disease ^13,14^. Glycosylation abnormalities represent an overt source of potential biomarkers for the diagnostic, prognostic, and treatment monitoring of various human diseases, including autoimmune, congenital, oncological, and neurodegenerative pathologies ^5,7,12^. In the emerging field of glycomedicine, novel therapeutic drugs or vaccines targeting tumor-associated carbohydrate antigens, like Lewis antigens and polysialic acids, are also currently in clinical evaluation ^5,15,16^.

Single-cell omics is a multi-faceted field that can potentially answer a myriad of questions in biomedical and clinical or fundamental biology applications ^17–19^. The commonly used approaches of analyzing populations of thousands to millions of cells in bulk samples may not reflect the cellular heterogeneity and hide subtle cell ome variabilities. On the contrary, the analysis of single cells may result in the characterization of distinct cell subpopulations and rare cells, and in the differentiation of various cell states, which are often overlooked in bulk sample analyses ^17,18^. Single-cell analysis is also beneficial to certain applications (e.g., minimally invasive liquid biopsy/microbiopsy-based cancer diagnosis and monitoring), where the amounts of original biological material may be limited for downstream molecular profiling. Single-cell omics encompasses a broad spectrum of analytical techniques that rely on vastly different technological principles, including genomics, transcriptomics, metabolomics, lipidomics, proteomics, and glycomics ^17,20,21^. In contrast to single-cell genomics and transcriptomics, single-cell proteomics and glycomics remain in their infancy. The analysis of proteomes and glycomes at the single-cell level is limited by the minute amounts of starting biological material and the inability to directly amplify protein and glycan species. For single-cell proteomics, ultra-sensitive mass spectrometry (MS)-based analytical platforms are under development ^22–25^. The Slavov group developed Single Cell ProtEomics by Mass Spectrometry (SCoPE-MS) techniques that rely on liquid chromatography (LC)-MS/MS methods with tandem mass tag (TMT)-labeling of peptides and a carrier channel for quantifying protein covariation across hundreds and potentially thousands of single cells ^26,27^. The Kelly ^28,29^ and Mechtler ^30^ groups succeeded in developing reliable platforms for label-free single-cell proteomics using in-house or commercial nanoliter-liquid handlers. Gebreyesus et al. implemented a fully automated workflow combining microchips and MS for sample preparation and bottom-up analysis, which resulted in the detection of > 1,500 protein groups from one single mammalian PC-9 cell ^31^. Our group recently reported a capillary electrophoresis (CE)-MS method for top-down single-cell proteomic profiling ^32^. This proof-of-concept approach allowed us to identify > 60 unique proteoforms in single HeLa cells ^33^.

Contrary to the rapidly growing field of single-cell proteomics, single-cell glycomics (SCG, which is a new suggested abbreviation) demonstrated lagging progress. This may be explained by i) the substantially lowered amounts of biological material available (glycosylation may account for only ~ 1–10% of the total mass of one human glycoprotein ^1^), and ii) the necessity to label the native glycans in the most commonly used positive electrospray ionization (ESI)-MS mode for increased ionization efficiency and detectability of released glycans, which obviously induces additional and substantial sample losses during the sample processing. Analytical platforms were implemented for total cellular glycome analysis of various mammalian cells, but these studies required large amounts of cells (~ 10^5^–10^7^ cells) ^34,35^. To date, only a few analytical technologies have been developed for SCG. The Johnston group developed the SUrface-protein Glycan And RNA-seq (SUGAR-seq) method for the analysis of the transcriptome, extracellular epitopes, and N-linked glycosylation at the single-cell level, using biotinylated lectins and anti-biotin antibodies combined with multimodal RNA-seq technology ^36^. Oligonucleotide-labeled lectins were used by the Tateno group for sequencing-based glycomic profiling of single cells, which enabled the acquisition of 39 lectin-binding signals per single cell and provided an informative picture of cell surface glycosylation ^37^. Roan and co-workers implemented a cytometry time-of-flight-lectin (CyTOF-Lec) technique for the simultaneous detection and quantification of proteins and glycans on the surface of human cells ^38^. In this approach, lanthanide-conjugated lectins were combined with traditional CyTOF mass cytometry using lanthanide-conjugated antibodies to specifically bind glycans and proteins and quantify them (through the lanthanide metal quantification) using inductively-coupled-plasma-MS (ICP-MS). The developed technique showed that CD4 + T cell surface glycosylation could influence the susceptibility of CD4 + T cells to viral infection. Nevertheless, the above-described approaches involve tedious, expensive, and time-consuming analytical workflows with sophisticated instrumentation and, most importantly, do not allow the direct analysis, quantitation, and accurate structural characterization of the glycans. Furthermore, some cell surface glycans may not interact with the lectins selected in the developed methodologies. These last few years, computational modeling software tools to predict the glycome at the single-cell level were also developed, based, for example, on single-cell RNA-seq transcriptomics data ^39^. Yet, so far, methods enabling the direct analysis, characterization, and quantitation of cell glycomes at the single-cell level have not been reported yet.

In this work, we developed an in-capillary sample processing methodology coupled with high-sensitivity label-free CE-MS for native N-glycan profiling of minute amounts of biomedically-relevant specimens and single mammalian cells. Blood-derived isolates (serum IgM and IgG, total plasma, and plasma extracellular vesicles (EVs)), and mammalian cells (HeLa and U87) were loaded and processed in the CE capillary for N-glycan release with PNGase F prior to CE-MS analysis. The mild conditions used for N-glycan release allowed us to preserve the cell membrane integrity and specifically liberate cell surface N-glycans. As in our previous work ^40^, N-glycans were analyzed in their native underivatized state to preserve their endogenous glycan features and eliminate the drawbacks associated with any labeling procedures, including incomplete derivatization, side-products, sample losses during cleanup steps, and high levels of defucosylation/desialylation during sample preparation and MS analysis. For glycan analysis of intact mammalian cells (1–10 cells), the manual hydrodynamic cell loading procedure described in our previous work ^32^ was further optimized not only to increase the robustness and throughput of cell loading, but also to improve the detectability and separation of the released N-glycans during CE-MS analysis. The vastly simplified in-capillary sample preparation approach coupled online with CE-MS eliminated sample losses associated with sample handling and transfer steps in the offline approach and allowed us to analyze sub-ng-levels of model proteins and pL-levels of total plasma, as well as single mammalian cells. To the best of our knowledge, such an approach enabling direct analysis and quantification of N-glycans derived from one single cell has not been reported yet. In addition, biochemical stimulation of mammalian cells induced significant qualitative and quantitative changes in the treated cells’ glycosylation profiles and confirmed the potential of the method to detect cell glycome alterations in biological and biomedical applications at the single-cell level.

## METHODS

### Materials and chemicals.

Deionized water, methanol (99.9%, LC/MS Grade), Gibco F12-K medium, Gibco DMEM medium, Gibco 0.25% Trypsin-EDTA, Gibco penicillin-streptomycin (P/S), phosphate-buffered saline (PBS), CellMask^™^ plasma membrane stain (green), LIVE/DEAD^™^ fixable green dead cell stain kit, and lipopolysaccharide (LPS) were obtained from Thermo Fisher Scientific (Waltham, MA). 1 N NaOH, 1 N HCl, 5 N ammonium hydroxide, glacial acetic acid (99.99%), ultra-high purity ammonium acetate (99.999%), trypan blue, and total human serum IgM and human serum IgG isolates (purity ≥ 95%, based on non-reduced SDS-PAGE and verified by nanoLC-MS/MS of tryptic digests) were purchased from Sigma-Aldrich (St. Louis, MO). PNGase F enzyme was from New England Biolabs (Ipswich, MA). Fetal bovine serum (FBS) was purchased from R&D Systems (Minneapolis, MN). Platelet-free anticoagulated with EDTA pooled total human plasma (from blood donated by self-declared healthy male donors of 23–67 years old) was kindly provided by Prof. Ghiran’s laboratory (BIDMC, Boston, MA). All bare fused silica (BFS) capillaries (91 cm × 30 μm i.d. × 150 μm o.d.) with sheathless CESI-MS emitters in OptiMS^™^ cartridges were from SCIEX (Redwood City, CA). Aquapel^®^ was purchased at Pittsburgh Glass Works (Pittsburgh, PA).

### Cell culture.

HeLa-S3 and U87-MG cell lines (called HeLa and U87 cells thereafter) were from ATCC (Manassas, VA). HeLa cells were cultured in suspension at 37°C in a complete F-12K medium supplemented with 10% FBS, 1% P/S, and 5% CO_2_. The cell density was maintained within a range of 2 × 10^5^ – 1 × 10^6^ cells/mL. Adherent U87 cells were cultured at 37°C in DMEM medium supplemented with 10% FBS, 1% P/S, and 5% CO_2_. Upon reaching confluence, one flask of U87 cells was split into five flasks. Cells were stained with trypan blue and counted using a 2-chip disposable hemocytometer (Bulldog Bio, Portsmouth, NH) to estimate the cell density and viability.

### LPS treatment of HeLa and U87 cells

2 or 4 μL of 2.5 mg/mL LPS were added to the 5 or 10 mL culture media in each HeLa or U87 cell culture flask, to get a final LPS concentration of 1 μg/mL. The HeLa and U87 cells were exposed to LPS for 24 h before being harvested and analyzed.

### Cell pellet collection.

HeLa and U87 cells were collected, washed, counted, and subsequently centrifuged into pellets prior to CE-MS analysis. The HeLa cell pellets were obtained by direct centrifugation of the HeLa cell culture suspension at 300 × g for 5 min. To detach the U87 cells from the flask bottom, 0.25% trypsin-EDTA was added, followed by incubation at 37°C for 5 min. The digestion was stopped by adding complete DMEM medium, and the detached U87 cells were centrifuged at 300 × g for 5 min to obtain the cell pellets. HeLa and U87 cell pellets were washed three times with 1x PBS, and their viability and density were assessed using a 2-chip disposable hemocytometer prior to the final centrifugation at 300 × g for 5 min. The cell viability was typically > 90%. The cell pellets were kept on ice until their use.

### Offline cell loading into the CE capillary.

One or five cells were loaded offline into the silica surface OptiMS Cartridge, following the protocol described in our previous work ^32^, with modifications. The cell loading process was visualized and monitored under an IX83 microscope (Olympus, Center Valley, PA), using a 10x magnification. First, the inlet of the CE capillary separation line was immobilized on a glass slide (pretreated with Aquapel^®^) placed under the microscope. Then, the capillary inlet was immersed in a 40 μL droplet of 1 mM ammonium acetate pH 6.7. A hydrodynamic flow was generated by manually lifting or lowering by ~ 45 cm the electrospray emitter tip of the capillary (separation line outlet) to generate an ultra-low flow rate of ~ 140 pL/s and enable precise control of the cell influx. Flow towards the separation line inlet was created by lifting the emitter tip to expel air bubbles before cell loading or dislodge unwanted cells after cell loading. For cell loading, 5 μL of a cell suspension at ~ 5 cells/nL was mixed with the droplet in which the separation line inlet was immersed, while the emitter tip of the capillary was held at the same height as the separation line inlet to prevent any forward or backward flow. The cell-containing droplet was gently agitated with a pipet tip until a target single cell (e.g., with the desired size and morphology) was observed in close proximity to the inlet. Then, the emitter tip of the capillary was lowered to introduce the cell into the capillary, and the flow was maintained until the cell was located approximately 500 μm away from the capillary inlet for targeted cell injection. The same procedure was repeated several times to load manually the desired number of cells.

### Cell staining and visualization.

To record the cell morphology and size distribution, 5 μL of a suspension of unstained cells in 1x PBS (with a cell density of ~ 1 × 10^6^ cells/mL) were deposited on a glass slide and imaged with the microscope under bright field at 10x magnification. For improved visualization of the cell morphology and membrane integrity, the cells were stained with CellMask^™^ plasma membrane green stain, following a procedure adapted from the manufacturer’s protocol. The 1,000x concentrated stain solution was diluted to 1x working solution with PBS. Subsequently, the cell pellet was resuspended with the working solution to an approximate cell density of 1 × 10^6^ cells/mL. Then, the cells were incubated in the dark for 30 min, followed by three washes with PBS to remove the excess stain. For fluorescence microscopy imaging of stained cells loaded within the capillary, the polyimide coating was removed before the experiments to avoid interference. To determine the cell viability and membrane integrity under the selected in-capillary sample processing conditions (i.e., after 60 min of incubation with the PNGase F enzyme in 1 mM ammonium acetate pH 6.7 buffer), the cells were stained with LIVE/DEAD^™^ fixable green dead cell stain. For this, one vial of the fluorescent dye was resuspended with 50 μL of DMSO. HeLa cells were harvested, washed, and resuspended with 1 mM ammonium acetate pH 6.7 to an approximate cell density of 5 × 10^5^ cells/mL. Then, 1 μL of the resuspended dye was added to 1 mL of the cell suspension. Finally, 20 μL of the stained cells were mixed with 15 mIU of PNGase F. Bright field and fluorescence-based microscopic images were acquired at different time points to evaluate the cell viability and morphology during the deglycosylation step with PNGase F.

### Preparation and characterization of EV isolate

Plasma-derived EVs were isolated using a size exclusion chromatography (SEC) column with a Sepharose CL-2B stationary phase. Briefly, 100 μL of platelet-free anticoagulated with EDTA pooled human plasma (from blood donated by self-declared healthy male donors of 23–67 years old) were loaded on the SEC column. EVs were eluted from the SEC column with 0.1 × dPBS, and the EV-containing fractions were pooled. The pooled EV fractions were then concentrated using Amicon^®^ 30 kDa MWCO ultrafiltration devices to a final volume of ~ 33 μL, and stored at 4°C until their analysis. The approximate EV particle concentration was estimated to be 1 × 10^10^ EV particles/mL, based on a combination of EV counting, using tunable resistive pulse sensing (TRPS), nano-flow cytometry, and immunoaffinity-based interferometry.

### In-capillary sample processing and CE methods.

In-capillary sample processing for N-glycan release with PNGase F and CE-MS experiments were conducted using a CESI 8000^™^ instrument (SCIEX). In all experiments, bare fused silica (BFS) OptiMS capillaries (91 cm × 30 μm i.d. × 150 μm o.d.) were used. Prior to each online or offline sample injection, a series of rinses of the separation and conductive lines were performed. For the separation capillary, these rinses included: MeOH (100 psi, 10 min), 0.1 M NaOH (100 psi, 3 min), 0.1 M HCl (100 psi, 3 min), and Milli-Q water (100 psi, 5 min), followed by the background electrolyte (BGE) (100 psi, 7 min). The conductive line was rinsed with the BGE (100 psi, 2 min). Before and after online (model glycoproteins, whole plasma, EVs, and ~ ten cells (referred to as “bulk cells” in this study)) or offline (1–5 cells) sample loading into the CE capillary inlet, a plug (1 or 2 nL applying 1 psi for 6 or 12 s) of a PNGase F digestion solution at 1.1 mIU/μL in 7 mM NaCl, 3 mM Tris-HCl, and 0.7 mM Na_2_EDTA was injected into the capillary using the CESI 8000 instrument. For offline cell loading, the CE cartridge was removed from the CESI instrument after the injection of the first PNGase F plug for manual cell loading, as described above, and placed back in the CESI instrument for subsequent in-capillary sample processing. After online or offline sample loading, a short plug of water (1 nL) was injected before the injection of the second PNGase F plug, followed by a short plug (0.5 or 1 nL) of 50 mM ammonium acetate pH 6.7. Then, two voltage pulses of 20 kV were applied in normal and reverse polarity for 30 s with the BGE composed of 10 mM (ionic strength) ammonium acetate pH 4.5 with 10% isopropanol, before incubating the capillary inlet in a vial containing 50 mM ammonium acetate pH 6.7 for either 30 min (model glycoproteins, whole plasma, and EVs) or 60 min (mammalian cells). After the in-capillary incubation step (performed at ~ 12°C) for N-glycan release with PNGase F, a BGE plug (10 psi for 10 s (model glycoproteins and whole plasma) or 10 psi for 60 s (mammalian cells)) was injected in the capillary prior to label-free CE-MS analysis of released N-glycans performed as described below. All CE methods employed 20 kV in reverse polarity with a voltage ramp time of 1 min. The CE-MS experiments were carried out with a BGE of 10 mM (ionic strength) ammonium acetate pH 4.5 with 10% isopropanol. This BGE generated a relatively low cathodic EOF (μ_EOF_ 2.02 × 10^−8^ m^2^/V/s) based on the detection of a neutral marker (acetaminophen). All CE-MS analyses were performed with a CE supplemental pressure (SP) of 5 psi, which was applied 18 min after switching on the CE voltage at the beginning of the CE run. Due to the variability in the manually injected cell plugs (performed offline), the migration time ranges in CE-MS analysis of mammalian cells were normalized, based on the most abundant detected glycan species.

### For model glycoproteins

(IgM and IgG), isolated from blood serum by size-exclusion chromatography (IgM) and ion-exchange chromatography (IgG), sample injections were performed at 1 or 5 psi for 6 s, corresponding to 1 and 5 nL injection volumes, respectively (i.e., 0.16 and 0.8% of the capillary volume, respectively). Three replicate analyses were performed with the injection of 0.1 ng and 5 ng of IgM, corresponding to ~ 60 pL and ~ 3,000 pL of human serum, respectively. Three replicate analyses were performed with the injection of 0.5 ng and 5 ng of IgG, corresponding to ~ 50 pL and ~ 500 pL of human serum, respectively.

### For total plasma isolate

1 mL of whole blood plasma isolate was centrifuged at 16,000 × g for 20 min at 4°C, and the supernatant was carefully pipetted to avoid collecting the lipid layer. For CE-MS analysis of total plasma, sample injections were performed at 1 psi for 6 s, corresponding to 1 nL injection volumes, and 5, 50, or 500 pL of plasma, depending on the dilution of the “pre-processed” plasma sample in water.

### For EV isolate

sample injections were performed at 1 psi for 6 s (1 nL injected) or 5 psi for 60 s (50 nL injected), corresponding to injected amounts equivalent to ~ 3 nL and ~ 150 nL of plasma, respectively.

### For cell analysis

the cell pellets were resuspended in 200 μL of 1 mM ammonium acetate pH 6.7 to get a final cell density of ~ 5 cells/nL. For online cell loading of ~ 10 cells, 2 nL of a cell suspension at ~ 5 cells/nL were injected, applying 1 psi for 12 s. In the offline cell loading mode, 1 to 5 cells were selected and injected manually as described above, and the 1–5 cell-containing plugs corresponded to ~ 4–6 nL injection volumes, based on microscope visualization. Owing to cell size variations, sets of five repetitive analyses were systematically performed with one (offline injection), five (offline injection), and ~ ten (online “bulk sample” injection) mammalian cells for each cell type (HeLa and U87 cells). CE-MS analyses of a blank sample of water were performed systematically to confirm insignificant levels of carryover derived from the analysis of preceding biological samples (blood-derived isolates and mammalian cells). For single-cell analysis, a water blank sample was analyzed between each single-cell injection. CE-MS analyses of the cell suspension medium (i.e., 1 mM ammonium acetate cell suspension buffer) were also performed. For these control analyses, 2–4 nL of water or cell suspension medium were injected inside the capillary and processed using the developed workflow, including the digestion step with PNGase F.

### MS instrumentation and techniques.

The CE capillary was interfaced with an Orbitrap^™^ Fusion Lumos^™^ mass spectrometer using a Nanospray Flex ion source (both Thermo Fisher Scientific, Bremen, Germany). All analyses were carried out in negative ESI mode. The nanoelectrospray potential was set to −1.8 kV. The ion transfer tube (ITT) temperature was set to 150°C (the distance between the electrospray emitter tip and the MS ITT was set to ~5 mm). The CE-MS analyses were performed with automatic gain control (AGC) of 1 × 10^6^ or 250%, a maximum injection time of 250 ms, 5 microscans, an S-lens voltage set to 65 eV, the nominal resolving power of 120,000 at 200 *m/z*, and in-source collision-induced dissociation (ISCID) at 70 eV. For CE-MS^2^ experiments, instrument resolving power was set at 60,000 at 200 *m/z* with 1 microscan. AGC was set to 2 × 10^5^ with a maximum injection time of 1,000 ms. An isolation window of 2 *m/z* was selected, and 32 eV was determined to provide the optimum normalized collision energy. CE-MS^1^ was performed as described above.

### Data analysis.

For data acquisition and processing, Xcalibur^™^ (v. 3.1) software was used. CE-MS data were processed with GlycReSoft (v. 3.10) software (Boston University, Boston, MA, USA). Analyses of CE-MS^2^ data were performed with SimGlycan (v. 5.91) software (Premier Biosoft, Palo Alto, CA, USA). The generated results were based on the processing of three replicate (model proteins, total plasma, and EVs) and five repetitivee (mammalian cells) analyses. For CE-MS^1^ processing with GlycReSoft, a mass matching error tolerance of 20 ppm was used in all searches. Up to six charge states, and sodium and ammonium adducts were included in the search. Other parameters were the same as described in our previous reports ^40,41^. The glycan identification analysis of the CE-MS data was conducted using database searches against in-house built mammalian database (version of December 2020) encompassing 27,335 N-glycan compositions (the mammalian database provided with the GlycReSoft software package encompasses 1,766 N-glycan compositions). For CE-MS^2^ processing with SimGlycan, a 20 ppm precursor mass tolerance and a 10 ppm fragment mass tolerance were used in all searches. Non-labeled glycans (unmodified or with sodium adduction) were searched selecting the options “Underivatized” and “Free” in the chemical derivatization and reducing terminal windows, respectively. Other parameters were as described in our previous studies ^40,41^. The glycan composition identification results were mainly based on CE-MS data processing using GlycReSoft. As additional verification of the plausible glycan identifications made using GlycReSoft, several supplementary levels of manual data examination were applied according to our recent studies ^40,41^. In brief, this verification included 1. Predictable trends in CE-MS migration patterns, 2. Charge state and isotopic distributions characteristic to glycan ions, 3. Detection of neutral losses, and 4. Manual examination of CE-MS^2^ data for low intensity parent ions. The relative quantitation of the detected N-glycans was based on the single-stage MS signal intensities or peak areas of the detected N-glycans that were normalized with respect to the summed MS signal intensities or peak areas of all the N-glycans detected in the sample. In addition, a qualitative comparison was performed based on the fractional distributions corresponding to the number of specific species (e.g., diasialylated glycans) out of the total number of N-glycans detected and identified in the analyzed biological specimens.

The bar charts with individual data points, mean values, and error bars were plotted using the R language and ggplot2 package in the rStudio development environment (2023.03.0 + 386 “Cherry Blossom” Release). The R language in the rStudio development environment was also used to perform statistical ANOVA and paired t-tests. The open-access tBtools-II (v1.120) software ^42^ was employed to generate heatmap clustering, utilizing the Euclidean distance-based clustering method and the complete cluster approach. For data clustering, N-glycan abundances (based on peak intensities) were normalized with respect to the summed abundances of all the N-glycans detected in the analyzed biological sample, and the clustering was done using the normalized abundance values after imputing 10% of minimum abundance for missing values followed by log_2_ transformation. The PCA plots were created with the open-access version of ClustVis (https://biit.cs.ut.ee/clustvis/ software ^43^. The average cell diameters of HeLa and U87 cells were measured using the open-access ImageJ (v1.53k) software. The glycan structures were designed with the open-access version of GlycoWorkBench (v2.0). Other schematic images (e.g., cell structure illustration) were built with the BioRender graphical tool.

For the Euclidean distance-based hierarchical clustering of single HeLa and single U87 cells before LPS treatment, glycans that were detected in at least three CE-MS analyses out of the ten total repetitive analyses (i.e., five repetitive analyses for single HeLa cells and five repetitive analyses for single U87 cells) were selected. This selection generated a set of 47 glycans highly representative of single HeLa and single U87 cells. For the Euclidean distance-based hierarchical clustering of single HeLa and single U87 cells after LPS treatment, glycans that were detected in at least two CE-MS analyses out of the five repetitive analyses of LPS-treated HeLa cells, and glycans that were detected in at least two CE-MS analyses out of the five repetitive analyses of LPS-treated U87 cells were selected and added to the above-decribed 47 glycans that are highly representative of HeLa and U87 untreated cells. For the PCA analysis of the glycans identified in single HeLa and single U87 cells after LPS treatment, more stringent parameters were used. In this case, glycans that were detected in at least three CE-MS analyses out of the five repetitive analyses of LPS-treated HeLa cells, and glycans that were detected in at least three CE-MS analyses out of the five repetitive analyses of LPS-treated U87 cells were selected and added to the above-decribed 47 glycans highly representative of HeLa and U87 untreated cells.

## RESULTS

**Figure 1:**
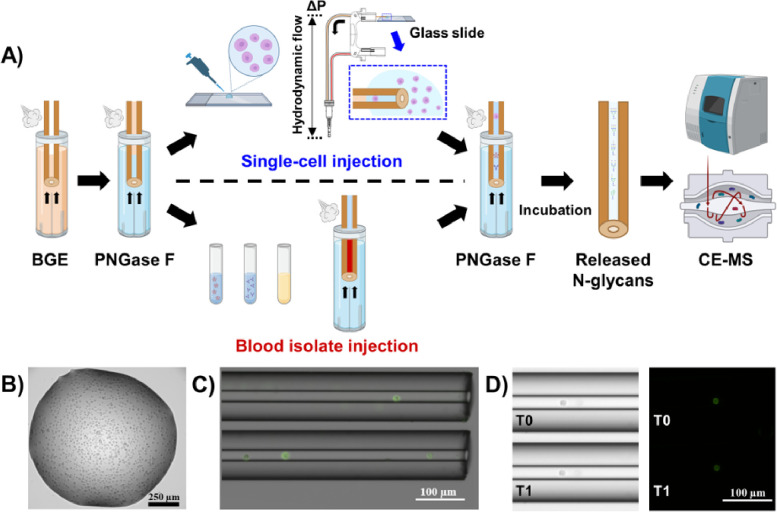
CE-MS-based experimental workflow for N-glycan profiling of single mammalian cells and blood-derived isolates. **A)** Schematic representation of the analytical platform developed for in-capillary sample processing and CE-MS analysis of N-glycans released from single mammalian cells and blood-derived isolates (model serum proteins, whole plasma, and plasma-derived EVs). Individual mammalian cells are injected manually using the height difference between the inlet and outlet ends of the CE capillary. The single-cell plug is sandwiched between two plugs (1 nL each) of a PNGase F digestion solution. After 30 min (blood-derived isolates) or 1 h (mammalian cells) incubation with PNGase F, the CE and MS electrospray voltages are triggered for label-free CE-MS analysis of released N-glycans. **B)** Representative image of a HeLa cell suspension droplet used for single-cell loading. **C)** Overlay of bright-field and fluorescence images showing one single HeLa (top view) and three HeLa (bottom view) cells loaded into the CE capillary. **D)**Comparative bright-field (on the left) and fluorescence (on the right) images displaying cell integrity before (T0) and after (T1) the in-capillary deglycosylation step with PNGase F.

We recently reported the first implementation of an in-capillary sample processing method coupled online with high-sensitivity CE-MS for top-down analysis of single mammalian cells and limited samples ^32^. The analytical workflow we previously developed for cell loading and in-capillary sample processing was adapted in the presented here study for CE-MS analysis of cell surface N-glycans with additional improvements. Besides, the non-labeling strategy for N-glycan analysis we recently developed ^40^, which demonstrated a sensitivity not yet reported for N-glycan profiling of scarce amounts of blood-derived isolates (sub-0.1 nL-level), was selected and optimized for CE-MS analysis of N-glycans released from single mammalian cells after their injection into the CE capillary. Figure 1 depicts the analytical workflow we developed for N-glycan profiling of single cells as well as for limited amounts of blood-derived isolates or other biological samples. The injected samples were sandwiched between two plugs of PNGase F and incubated inside the capillary. After the digestion step, the CE and ESI-MS voltages were triggered for online CE-MS analysis of the released N-glycans in their native non-labeled state. We started with the analysis of simpler sample types, moving on to more challenging samples.

### N-glycome profiling of blood-derived isolates

#### N-glycan profiling of human serum IgM.

**Figure 2:**
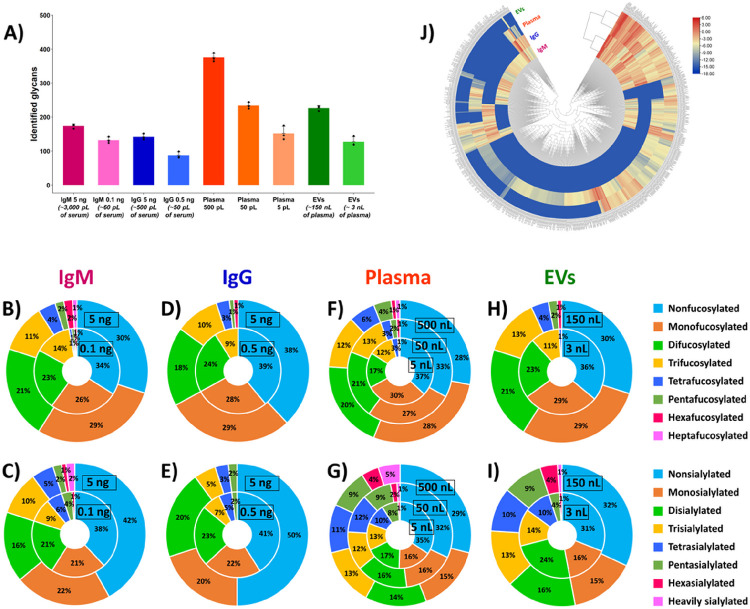
CE-MS-based N-glycan profiling of human blood-derived isolates. **A)** Number of N-glycans (n=3) identified in the four analyzed blood-derived isolates (human serum IgM, human serum IgG, total plasma, and EV isolate). **B-H)** Fractional distributions of fucosylated N-glycans detected in IgM (B), IgG (D), total plasma (F), and EVs (H). **C-I)** Fractional distributions of sialylated N-glycans detected in IgM (C), IgG (E), total plasma (G), and EVs (I). **J)**Euclidean distance-based hierarchical clustering of quantitative glycomic profiles of IgM, IgG, total plasma, and EVs, using injected amounts of 5 ng of IgM, 5 ng of IgG, 500 pL of plasma, and 50 nL of EV isolate (corresponding to ~150 nL of plasma), respectively. Red, yellow, and light blue colors correspond to high, medium, and low relative abundances based on the normalized N-glycan signal intensities. N-glycans that are not detected in the blood-derived samples are highlighted in dark blue.

Human immunoglobulin M (IgM), a heavily glycosylated multimeric protein (Mr_th_ 970 kDa and 1,080 kDa for pentameric and hexameric forms, respectively), was selected to develop and optimize the in-capillary sample processing method for N-glycan release coupled online to label-free CE-MS analysis. N-glycans account for ~10% of the total mass of IgM ^44^, and the serum level of IgM is in the range 0.4–2.5 mg/mL ^45,46^. CE-MS analysis of IgM-derived in-capillary released N-glycans resulted in the identification of 173 ± 6 (n = 3) non-redundant N-glycan compositions in human serum IgM isolate for injected amounts of 5 ng (i.e., 5 fmol) of protein, corresponding to ~ 500 pg of N-glycans and equivalent to the amount of IgM isolated from ~ 3 nL of human serum (Fig. 2A). Interestingly, the injection of sample amounts as small as 0.1 ng (i.e., 100 amol) of IgM, corresponding to ~ 10 pg of N-glycans and equivalent to ~ 60 pL of human serum, resulted in the detection and identification of 132 ± 9 (n = 3) N-glycans (Fig. 2A). Considering the current developments in single-cell analysis, it is worth noting that these minute amounts of proteins and glycans (~ 100 pg and ~ 10 pg, respectively) are equivalent to the protein and glycan content of one single mammalian cell ^20^. Using our newly developed label-free CE-MS-based workflow, the number of identified N-glycans was increased ~7-fold, compared to previously reported studies focused on N-glycan profiling of human serum IgM ^47,48^. Figure 2BC displays the fractional distributions of fucosylated and sialylated N-glycans identified in IgM, using 0.1 ng and 5 ng injected amounts of IgM. While highly fucosylated (up to 6 fucose residues) and highly sialylated (up to 6 sialic acid (SiA) residues) N-glycans were detected in the CE-MS analyses performed with either 5 ng or 0.1 ng of IgM, heptafucosylated and heavily sialylated (7–11 SiA residues) N-glycans were detected only with 5 ng of IgM injected amounts, indicating the very low abundances of these uncommon glycans.

We noticed that the developed and optimized workflow was well adapted to the analysis of 5 ng and sub-ng amounts of serum IgM. Using these tiny sample amounts and intensive rinsing steps between runs, no significant carryover derived from the analysis of preceding IgM samples was observed, based on the analysis of the water blank control sample (see Methods section). The injection of larger amounts of protein (e.g., 25–100 ng), which could potentially increase the glycan coverage of IgM, would require the re-optimization of several parameters, including the glycosidase: protein substrate ratio, the incubation time, and the rinsing steps between runs to efficiently clean the capillary. This scale-up workflow would obviously increase the sample processing and total analysis times. Since the goal of our study was to develop an effective and quick CE-MS-based workflow applicable to single-cell analysis, we estimated that glycan amounts released from the digestion of 0.1 to 5 ng of model glycoprotein within the CE capillary should reflect well the amounts of glycans released from one to ten mammalian cells.

#### N-glycan profiling of human serum IgG.

We tested the newly developed N-glycan profiling workflow with the CE-MS analysis of human IgG (Mr_th_ 150 kDa), another class of immunoglobulin less glycosylated than IgM. N-glycans account for ~ 2% of the total mass of IgG, and the human serum level of IgG is in the range 7–16 mg/mL ^45,46^. The developed CE-MS method allowed us to identify 142 ± 9 (n = 3) non-redundant N-glycan compositions in human serum IgG isolate for injected amounts of 5 ng (i.e., 33 fmol) of protein, corresponding to ~ 100 pg of N-glycans and equivalent to the amount of IgG isolated from ~ 500 pL of serum (Fig. 2A). Compared to our previous studies reporting N-glycan profiling of IgG ^40,41^, the injected amounts of IgG were decreased ~ 5-fold. With such low injected amounts, the number of identified N-glycans still largely exceeded (~ 6-fold) the number of N-glycans reported in human serum IgG by other groups ^49,50^. As observed for IgM, the injection of protein amounts larger than 5 ng of IgG, in order to increase the glycan coverage of IgG, would require a workflow re-optimization. We deemed it more relevant to decrease the injected protein amount to mimic the amount of glycans released from one single cell. The injection of ~ 0.5 ng (i.e., 3 fmol) of IgG resulted in the identification of 88 ± 10 (n = 3) N-glycans (Fig. 2A). These scarce amounts of IgG correspond to only ~ 10 pg of N-glycans and isolates from ~ 50 pL of serum. Figure 2DE displays the fractional distributions of fucosylated and sialylated N-glycans identified in IgG. In our previous work ^40^, we showed that hexa- and heavily sialylated N-glycans could not be detected in CE-MS analysis of non-labeled IgG-derived N-glycans using injected amounts equivalent to ~ 25 ng of IgG. As expected, glycans with a degree of sialylation ≥ 6 were not detected in such low 0.5–5 ng IgG sample amounts using the presented here workflow, since even larger injected amounts of serum IgG also did not allow us to detect them previously.

#### N-glycan profiling of total human plasma

Our developed workflow allowed us to analyze sub-nL volumes of total human plasma isolate, and enabled direct online N-glycan profiling of plasma volumes as small as 5 pL, which was not reported before. Data processing of CE-MS analyses resulted in the identification of 375 ± 12, 234 ± 10, and 152 ± 21 (n = 3) non-redundant N-glycan compositions in whole blood plasma for injected amounts of 500 pL, 50 pL, and 5 pL of plasma (i.e., ~ 1,500, 150, and 15 pL of human blood), respectively (Fig. 2A). In our previous work ^40^, 210 ± 12, and 62 ± 31 N-glycans were identified in whole plasma for injected amounts equivalent to ~ 160 pL and ~ 80 pL of plasma, respectively, using label-free CE-MS analysis of plasma-derived glycans released offline. The presented here results, therefore, demonstrate the superior performance of the newly developed in-capillary sample processing-based workflow for straightforward and unbiased N-glycome analysis of minute amounts of physiological fluids. As shown in Fig. 2FG, hexa- and heptafucosylated, and heavily sialylated (i.e., ≥ 7 SiA residues) N-glycans were not detected using ~ 5 pL of plasma injected volumes. These highly fucosylated and sialylated N-glycans were only detected using larger plasma volumes. Glycans containing up to 13–14 SiA residues were detected with the injection of 50 and 500 pL of plasma.

#### N-glycan profiling of blood-derived extracellular vesicles.

The developed in-capillary workflow was applied to the analysis of N-glycans released from human plasma-derived extracellular vesicles (EVs), another attractive source of disease biomarkers ^51,52^. Experiments were carried out with the injection of a purified EV isolate, containing ~ 1 × 10^4^ EV particles/nL (see Methods section). CE-MS analysis resulted in the detection and identification of 127 ± 14 and 226 ± 7 N-glycans in the total EV isolate, using 1 nL and 50 nL of EV isolate injection volumes, respectively (containing approximately 1 × 10^4^ EVs and 5 × 10^5^ EVs, respectively) (Fig. 2A). These injected amounts are equivalent to the EV content of ~ 3 nL and ~ 150 nL of plasma, respectively. Figure 2HI displays the fractional distributions of fucosylated N-glycans detected in the total EV isolate using injection volumes of 1 nL and 50 nL of the EV isolate (i.e., ~ 3 and ~ 150 nL of plasma equivalents). Interestingly, the injection of volumes as small as 1 nL of EV isolate resulted in the detection of tetrafucosylated and hexasialylated N-glycans. The injection of larger volumes of EV isolate (i.e., 50 nL) allowed us to increase the coverage of fucosylated glycans with the detection of penta- and hexafucosylated N-glycans, which were not detected using 1 nL of EV isolate. The injection of 50 nL of EV isolate also resulted in the detection of heavily sialylated N-glycans (up to 14 SiA residues), undetectable using 1 nL volumes of EV isolate. Compared to our previous study ^40^, a similar coverage of EV-derived N-glycans was achieved using the newly developed label-free CE-MS technique, based on the number and types (i.e., degrees of fucosylation and sialylation) of identified N-glycans for similar injected amounts. Finally, the presented here CE-MS-based workflow (similarly to our previously reported label-free CE-MS method ^40^) allowed us to further expand the catalog of blood-derived EV N-glycans, compared to other studies reported for N-glycome profiling of biofluid-derived EVs ^41,53^, using injected amounts as low as ~ 150 nL of plasma (i.e., ~ 400 nL of blood).

#### Differential N-glycan profiling of IgM, IgG, whole plasma, and EV isolates from blood.

A qualitative and quantitative comparative analysis of N-glycans detected in the four types of analyzed blood-derived isolates (IgM, IgG, total plasma, and EVs) was conducted with an exhaustive list of 679 glycans, encompassing all the non-redundant N-glycan compositions identified in the four blood isolates. This differential analysis further demonstrated the uniqueness and high complexity of the four examined N-glycomes (Fig. 2J and **Table S1**). Interestingly, and as expected, IgM and IgG immunoglobulins, and total plasma and plasma-derived EVs were clustered in two distinct clades, based on their respective N-glycome profiles (Fig. 2J). 68 N-glycans were uniquely detected in human serum IgM (**Figure S1**), among which 25% were highly fucosylated (i.e., 5–7 fucose residues) and 10% were highly sialylated (i.e., 5–11 SiA residues) N-glycans. 185 N-glycans were uniquely detected in total human plasma (**Figure S1**), among which 12% were highly fucosylated (i.e., 5–7 fucose residues) and 31% were highly sialylated (i.e., 5–14 SiA residues) N-glycans. In contrast, the numbers of N-glycans uniquely detected in human serum IgG and total EV isolate were relatively low (13 and 20 N-glycans, respectively, **Figure S1**). As expected based on our previous studies, unique IgG N-glycans did not exhibit high degrees of fucosylation and sialylation since, as described above, highly fucosylated and sialylated glycans were not detected in IgG. A few N-glycans unique to EVs were highly sialylated (i.e., 5–14 SiA residues) N-glycans. Similarities were observed in the relative abundance levels of the N-glycans detected in the four analyzed blood-derived samples (**Table S1**). Several glycans, including FA2G2S2, FA2BG2S2, A2G2S2, and A3G3S3, were detected at high abundance, and other glycans, including A4G4S4, FA4G4S4, A3G3S2, and FA3G3S2, were detected at medium abundance in each analyzed isolate sample. Other glycans, e.g., the set of fucosylated analogs of A3G3S3, exhibited different relative abundances in the four examined blood isolate types. As an illustration, FA3G3S3 was detected at lower abundance in the IgM and IgG isolates, compared to plasma and EVs. F2A3G3S3 was not detected in the IgG isolate and detected in relatively low abundance in the other three blood isolates. F3A3G3S3 was detected only in plasma and EV isolates at relatively low abundance, and F4A3G3S3 was detected only in total plasma. Several neutral glycans also exhibited significantly different abundance levels in the four types of the blood-derived isolates. For instance, Man10 was detected at much lower abundance in the IgM and EV isolates, compared to the IgG isolate and total plasma. Man9 was detected at higher abundance in the IgG isolate, compared to the other three blood isolates, while Man8 and Man7 were detected only in the IgG isolate (**Table S1**). Overall, the above-described results demonstrate that the newly developed workflow is a powerful, straightforward, flexible, and highly sensitive approach to decipher the N-glycomes of complex biological samples. It allowed us to further expand the glycan coverage of the four analyzed blood-derived isolates, including human plasma-derived EVs, using minute amounts of samples, and identify glycan species unique to a specific type of a blood isolate.

### Single mammalian cell N-glycome profiling

#### Single-cell loading and in-capillary N-glycan release.

Individual mammalian cells were introduced into the CE capillary in a controlled manner using a hydrodynamic injection mode (Fig. 1A and **Figure S2AB**), as described in the Methods section. To visually confirm the cell loading process, the cells were treated with a plasma membrane-binding dye and subsequently imaged using bright-field and fluorescence microscopy techniques (Fig. 1CD). Upon introduction into the capillary, the evaluated cells exhibited a tendency to weakly and transiently adhere to the capillary surface if the hydrodynamic flow was either halted or maintained at an excessively low rate. This phenomenon of cell immobilization is likely attributable to the formation of hydrogen bonds and van der Waals interactions between the silanol groups of the bare fused silica capillary surface and numerous chemical groups present on the cell surface. When the diameter of the injected cells was close to the internal diameter of the capillary, cells got slightly squeezed upon entering the capillary, and their size could further impede their mobility. This tendency was strategically exploited to facilitate cell stacking for the effective injection of several cells, as well as to better control the distance between the injected individual cells and the capillary inlet, thus making each injection more reproducible.

The cells loaded into the CE capillary were sandwiched between two plugs of a PNGase F digestion solution, and two short CE voltage pulses (30 s each) were applied in normal and reverse polarity to effectively mix the cells with the glycosidase (see Methods section). No lysis buffer was employed and/or injected to preserve the cell integrity and release only the N-glycans from the cell surface. Ideally, to preserve cellular integrity, the cells should be maintained in a buffer solution that closely mimics physiological pH and osmolarity. However, such buffers are typically incompatible with MS or CE analysis and may result in ionization suppression, adduct formation, and decreased separation performance phenomena. In this study, a stacking strategy was used to enhance the peak shape and intensity and improve the separation of the detected glycans. To enable this strategy, the cells were resuspended in 1 mM ammonium acetate pH 6.7, immediately prior to their loading into the CE capillary, and the commercial PNGase F enzyme was diluted 7-fold in water to highly decrease the salt concentration (see Methods section). Given that the cells were exposed to a low osmolarity environment during the deglycosylation step for N-glycan release, an assessment of the post-incubation cell integrity was conducted through the offline incubation of single cells for 1 h, using the conditions employed in the in-capillary sample processing workflow (i.e., the cells were sandwiched between two PNGase F plugs). Fluorescence imaging of the single cells prior to and after offline incubation in a small piece of capillary did not reveal discernible alterations in the cell morphology, size, or membrane integrity (Fig. 1D). To further check the cell viability, morphology, and membrane integrity under the selected in-capillary sample processing conditions, a suspension of HeLa cells stained with a fixable dead cell dye (see Methods section) was incubated for 1 h with PNGase F in 1 mM ammonium acetate pH 6.7. Based on microscopic visualization (**Figure S2EF**), we estimated that > 40% of the HeLa cells were still alive after the deglycosylation step with PNGase F. We also noticed that the majority of the dead cells exhibited a morphology very similar to that of the live cells, suggesting that the cell integrity could be preserved under our experimental conditions for N-glycan release. Therefore, we concluded that the majority of detected and identified glycans were removed from the cell surface in our in-capillary cell processing proof-of-concept experiments.

#### N-glycan profiling of single, five, and ~ ten HeLa cells.

**Figure 3:**
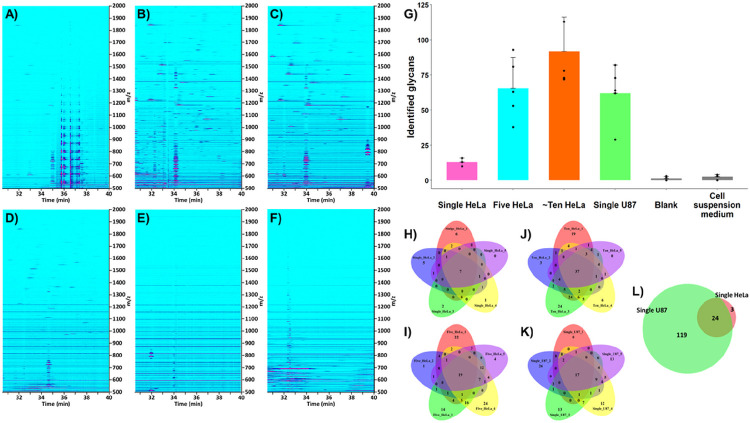
CE-MS-based N-glycan profiling of HeLa and U87 mammalian cells. **A-F)** Representative ion density maps acquired in label-free CE-MS analyses of N-glycans released from (A) one HeLa cell, (B) five HeLa cells, (C) ~ten HeLa cells, (D) one U87 cell, (E) water blank sample, and (F) cell suspension medium (a normalized intensity level of 3.9 × 10^4^ was selected for each density map). **G)** Number of N-glycans (n=5) identified in the analyzed mammalian cell and control samples. **H-K)** Venn diagrams illustrating the overlap of N-glycans identified in five repetitive analyses acquired with the injection of one (H), five (I), and ~ten (J) HeLa cells, and one U87 cell (K). **L)** Venn diagram illustrating the overlap of N-glycans identified in single HeLa and single U87 cells, based on the total number of N-glycans identified in HeLa and U87 single cells (see panels H and K, respectively).

To assess the capability of the developed workflow for direct and unbiased N-glycan profiling of mammalian cells, sets of five repetitive experiments were performed with the injection of one (**Figure S2A**), five, and ~ ten (i.e., 10 ± 3 cells, referred to as “bulk sample”, see Methods section) HeLa cells. Characteristic ion density maps acquired in CE-MS analysis of HeLa cell-derived N-glycans are presented in Fig. 3ABC. Processing of CE-MS^1^ data resulted in the identification of 13 ± 3 (one HeLa cell), 66 ± 22 (five HeLa cells), and 92 ± 24 (~ ten HeLa cells) N-glycans (n = 5), respectively (Fig. 3G). As expected, due to cell-to-cell heterogeneity (arising from, inter alia, molecular variability and cell-cycle position) and cell size variations (the surface areas of the injected HeLa cells being in the range 1,282–3,258 μm^2^, **Figure S2C**), the number and type (i.e., monosaccharide composition) of identified N-glycans exhibited significant variations. Figure 3HIJ depicts the overlap of the N-glycans detected in the five repetitive analyses acquired for each HeLa cell loading. In total, 27, 148, and 160 non-redundant N-glycan compositions were identified in one, five, and ~ ten HeLa cells, respectively. As shown in Fig. 3EG, the levels of carryover derived from the analysis of preceding HeLa cell samples were insignificant while using the described capillary rinse cycles, based on control analyses performed with a water blank sample (see Methods section). CE-MS analyses of the cell suspension medium, collected from the same cell suspension used to inject individual cells, were also carried out. These experiments allowed us to confirm that the detected glycans were not derived from extracellular proteins or contaminants present in the cell medium (Fig. 3FG).

**Figure 4:**
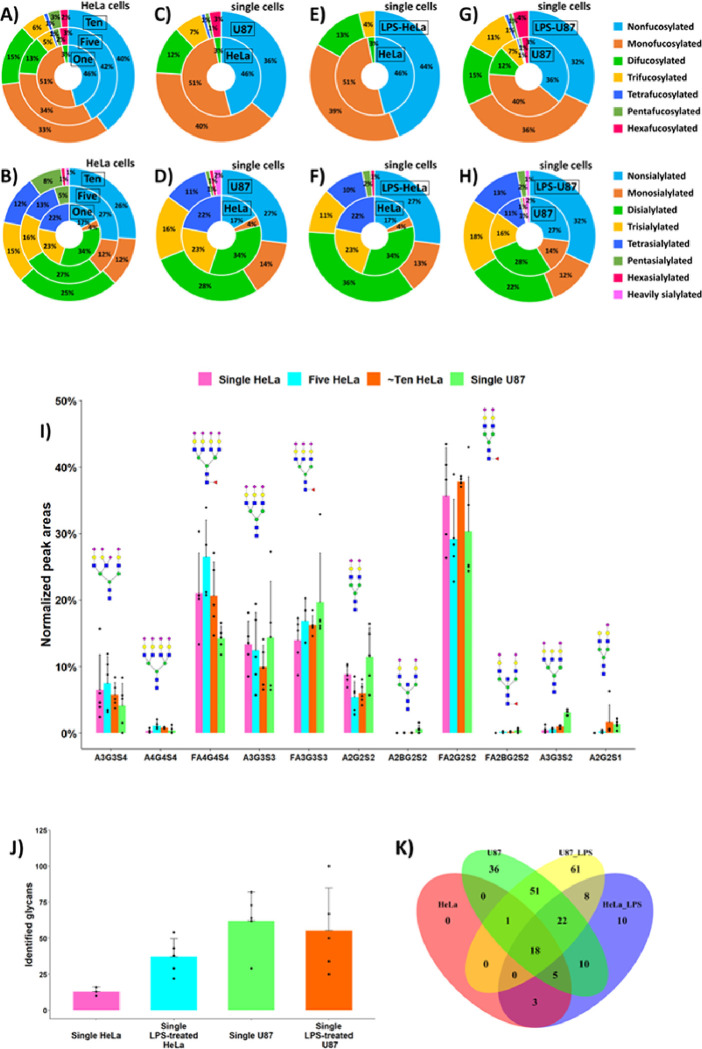
Fractional distributions of fucosylated and sialylated N-glycans detected in HeLa and U87 mammalian cells. **A-G)** Fractional distributions of fucosylated N-glycans detected in one, five, and ~ten HeLa cells (A), HeLa and U87 single cells (C), LPS-treated and untreated single HeLa cells (E), and LPS-treated and untreated single U87 cells (G). **B-H)** Fractional distributions of sialylated N-glycans detected in one, five, and ~ten HeLa cells (B), HeLa and U87 single cells (D), LPS-treated and untreated single HeLa cells (F), and LPS-treated and untreated single U87 cells (H). **I)** Normalized abundances of eleven representative N-glycans detected in one, five, and ~ten HeLa cells, and one U87 cell. Glycan symbols: blue square, GlcNAc; red triangle, Fuc; green circle, Man; yellow circle, Gal; purple diamond, Neu5Ac. **J)** Number of N-glycans (n=5) identified in single HeLa and single U87 cells before and after LPS stimulation. **K)** Venn diagram illustrating the overlap of N-glycans identified in single HeLa and single U87 cells with and without LPS treatment, based on the total number of N-glycans identified in the respective analyzed samples.

Higher levels of fucosylation (up to 6 fucose residues) and sialylation (5–12 SiA residues) were detected in 5–10 HeLa cells, compared to single HeLa cells, for which the degrees of fucosylation and sialylation of identified N-glycans did not exceed 2 and 4, respectively (Fig. 4AB). These results indicated the extremely low abundance levels of highly fucosylated and highly sialylated N-glycans in HeLa cells since they could not be detected at the single HeLa cell level using our developed workflow. Mono- and difucosylated N-glycans accounted for 51% and 3% of the total glycans detected in single HeLa cells, respectively (46% of glycans were nonfucosylated). For 5 and 10 HeLa cells, the fractional distributions of fucosylated N-glycans were 34%, 13%, 5%, 1%, 2%, and 3% (five HeLa), and 33%, 15%, 6%, 1%, 3%, and 2% (~ ten HeLa) for mono-, di-, tri-, tetra-, penta-, and hexafucosylated N-glycans, respectively (Fig. 4A). Mono-, di-, tri-, and tetrasialylated N-glycans accounted for 4%, 34%, 23%, and 22% of the total glycans detected in single HeLa cells, respectively (17% of glycans were nonsialylated). For 5 and 10 HeLa cells, the fractional distributions of sialylated N-glycans were 12%, 27%, 16%, 13%, and 5% (five HeLa), and 12%, 25%, 15%, 12%, 8%, 1%, and 1% (~ ten HeLa) for mono-, di-, tri-, tetra-, penta-, hexasialylated, and heavily sialylated (≥ 7 SiA residues) N-glycans, respectively (Fig. 4B).

As expected, due to cell-to-cell heterogeneity, high variations in the absolute glycan abundances measured in the five repetitive analyses were observed for one, five, and ~ ten injected HeLa cells (e.g., for single HeLa cell measurements, the relative standard deviations (RSD) of peak areas could be as high as 65%). We hypothesize that such significant variation might be mostly attributed to the cell size, surface area, and cell cycle state. Nevertheless, a substantial increase in the glycan abundance levels was demonstrated with increased loaded cell numbers. As an illustration, **Figure S3A** shows the summed absolute abundances of eight selected representative N-glycans, detected in high abundance in one, five, and ~ ten HeLa cells. A linear relationship was demonstrated between the injected cell number and the total cellular glycan amount for the eight selected glycans, based on peak area measurements (**Figure S3B**). Figure 4E displays the normalized abundances of eleven representative N-glycans detected in one, five, and ~ ten HeLa cells (i.e., each glycan abundance was normalized with respect to the summed abundances of the eleven selected glycans). FA2G2S2, A3G3S3, FA3G3S3, and FA4G4S4 were detected at high abundance levels in the analyzed HeLa cells. To the contrary, A2G2S1, A2BG2S2, FA2BG2S2, A3G3S2, and A4G4S4 were measured at low abundances. Overall, the relative abundances of the eleven selected HeLa cell-derived N-glycans were consistent across different cell loading levels, based on peak area measurements. For instance, the relative abundance of FA4G4S4 was approximately twice lower than that of FA2G2S2 but about three times higher than that of A3G3S4 in one, five, and ~ ten HeLa cells.

**Figure 5:**
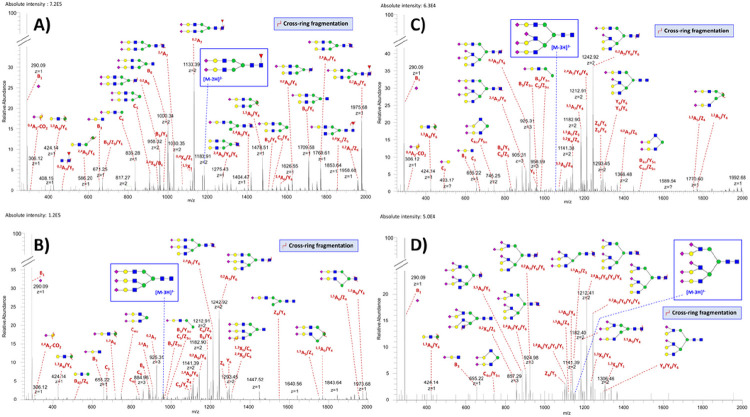
CE-MS2-based structural characterization of representative HeLa and U87 cell-derived N-glycans. **A-D)** Characteristic CE-MS^2^ spectra of (A) the fucosylated disialylated glycan **FA2G2S2**, (B) the trisialylated glycan **A3G3S3**, (C) the tetrasialylated glycan **A3G3S4**, and (D) the pentasialylated glycan **A3G3S5**, selecting the molecular ions at *m/z* 1,183.42, 958.66, 1,055.69, and 1,152.73 as precursor ions, respectively. Fragment ions are annotated based on the Domon and Costello nomenclature. Blue square, GlcNAc; red triangle, Fuc; green circle, Man; yellow circle, Gal; purple diamond, Neu5Ac. Symbol Ζ indicates cross-ring fragmentation. Only the most intense/relevant fragments are annotated in the shown spectra.

CE-MS^2^ analyses of HeLa cell-derived N-glycans were performed to confirm the N-glycan composition identification results and provide information on structural features of the detected glycans (e.g., antenna-branching, fucose position, and SiA linkage). CE-MS^2^ experiments performed with ~ ten HeLa cells resulted in accurate and unambiguous structural characterization of 53 N-glycans in HeLa cells, among which acidic (i.e., sialylated) and neutral glycans (**Table S2**). As expected, neutral glycans (mobilized under the applied electric field through ion-dipole interactions with acetate anions present in the BGE ^40^) migrated later than sialylated glycans. Figure 5 shows characteristic MS^2^ spectra of three representative sialylated N-glycans detected in HeLa cells. The molecular ions at *m/z* 1,183.42, 958.66, and 1,055.69 were selected as precursor ions for the MS^2^-based structural characterization of **FA2G2S2** (Mr_th_ 2,368.84 Da, Fig. 5A), **A3G3S3** (Mr_th_ 2,879.01 Da, Fig. 5B), and **A3G3S4** (Mr_th_ 3,170.11 Da, Fig. 5C), respectively. The MS^2^ spectra of these sialylated glycans all exhibited a predominant fragment ion B_1_^1−^ at *m/z* 290.09, corresponding to the loss of one SiA residue at the termini of the antennae, and the diagnostic ion ^0,4^A_2_-CO_2_^1−^ at *m/z* 306.12, revealing the presence of α−2,6 5-N-acetyl-neuraminic acid (Neu5Ac) linkages ^54^. In the mass spectra of the fucosylated FA2G2S2 glycan, the characteristic mass difference of 206.08 Da between ^2,4^A_7_^2−^ (*m/z* 1,029.85) and ^0,2^A_7_^2−^ (*m/z* 1,132.89), and ^2,4^A_7_/Y_6_^1−^ (*m/z* 1,769.61) and ^0,2^A_7_/Y_6_^1−^ (*m/z* 1,975.68) ion pairs located the fucose on the chitobiose core ^40,55^. For the tri-antennary A3G3S3 glycan, the diagnostic ions B_5_/Z_3α_^1−^ (*m/z* 961.31), B_5_/Y_3α_^1−^ (*m/z* 979.32), and C_4α_^2−^ (*m/z* 745.25) allowed us to assign a branched 3-linked antenna ^40,55^. Similarly, the fragment ions B_5_/Z_3α_^1−^ (*m/z* 961.31), B_5_/Y_3α_^1−^ (*m/z* 979.32), and C_4α_/Y_5α_^2−^ (*m/z* 745.25) were diagnostic of a branched 3-linked antenna for the tri-antennary A3G3S4 glycan. The pentasialylated glycan **A3G3S5**, a complex glycan that was rarely structurally characterized by tandem MS in the past ^40^, was also detected in HeLa cells (see below and Fig. 5D for the MS^2^ fragmentation pattern of this glycan). CE-MS^2^ data also allowed us to structurally characterize neutral N-glycans in HeLa cells (**Table S2**), including high mannose-type N-glycans in HeLa cells, from Man2 to Man13, with or without a bisecting GlcNAc residue (**Table S2**). **Figure S4** depicts the fragmentation patterns of Man6, Man7, and Man13. For these glycans, characteristic fragment ions (e.g., at *m/z* 179.06, 323.10, 485.15, 869.27, and 1,031.33) were supportive of a branched oligomannosyl structure ^56^.

#### Differential N-glycome analysis of single HeLa and U87 cells.

Next, the developed in-capillary workflow was applied to the CE-MS analysis of single U87 cells to assess, as a proof-of-concept, whether we could detect qualitative and/or quantitative differences in cell surface N-glycomes of different cell types at the single-cell level. In comparison to single HeLa cells, a significantly higher number (~ 5-fold) of N-glycans were detected and identified in single U87 cells. The five repetitive experiments carried out with single U87 cells (**Figure S2B**) resulted in the detection of 62 ± 20 N-glycans per single cell (Fig. 3DG). In total, 143 non-redundant N-glycan compositions were identified in the analyzed single U87 cells (Fig. 3K). The examination of HeLa and U87 cell-containing droplets under a bright-field microscope showed that the two cell lines exhibited similar morphological characteristics in suspension but distinct size distributions. The average diameters and surface areas of representative HeLa and U87 single cells were determined to be 21.8 ± 4.9 μm and 1,563 ± 763 μm^2^ (HeLa), and 26.1 ± 6.6 μm and 2,282 ± 1,266 μm^2^ (U87), respectively (see Methods section and **Figure S2CD**). The higher number of N-glycans detected in single U87 cells could, therefore, be attributed in part to the larger size of this cell type (~ 2-fold higher surface area), compared to HeLa cells. Yet, the non-linear relationship between the number of detected glycans and the size of the analyzed mammalian cells indicated that, as expected, other factors, e.g., unique molecular features and their abundances specific to HeLa and U87 cells, contributed to the significantly different numbers of N-glycans detected at the surface of HeLa and U87 single cells. Besides, the intrinsic cell morphology and structural characteristics of each cell type might also create locus-dependent steric hindrances at the surface of the single cells, resulting in differential accessibility of PNGase F to these specific cell surface loci. We expect to observe the increased depth of N-glycome profiling at the levels of five and ten U87 cells, similarly to the determined trends in the discussed above HeLa experiments. However, examining these trends experimentally was not the focus of this proof-of-concept analysis of U87 cells.

89% of the glycans identified in single HeLa cells were also detected in single U87 cells (Fig. 3L). These commonly detected glycans accounted for 17% of the total number of N-glycans identified in single U87 cells, indicating that 83% of the glycans detected in U87 cells were specific to this cell line or could not be detected in HeLa cells due to their very low abundance or under-representation in single HeLa cells. In single U87 cells, higher levels of fucosylation (up to 6 fucose residues) and sialylation (up to 12 SiA residues) were detected, compared to single HeLa cells (Fig. 4CD). Consequently, the unique glycans detected in single U87 cells encompassed tri-, tetra-, penta-, and hexafucosylated glycans, and sialylated glycans composed of 5–12 SiA residues, which were not detected in single HeLa cells. In single U87 cells, mono-, di-, tri-, tetra-, penta-, and hexafucosylated N-glycans accounted for 40%, 12%, 7%, 1%, 1%, and 3%, respectively (36% of glycans were nonfucosylated, Fig. 4C). The fractional distributions of sialylated N-glycans were as follows: 14% (mono-), 28% (di-), 16% (tri-), 11% (tetra-), 1% (penta-), 1% (hexa-), and 2% (heavily sialylated, i.e., ≥ 7 SiA residues), respectively (27% of glycans were nonsialylated) (Fig. 4D).

Noticeable differences in the abundances of the N-glycans detected in HeLa and U87 single cells were also observed, based on peak area measurements. As an illustration, Fig. 4I shows the relative abundances of eleven selected N-glycans commonly detected in HeLa and U87 single cells. Based on the statistical paired t-test (**Table S3AB**), a couple of glycans were detected in significantly higher or noticeably higher abundance levels in U87 cells, e.g., FA2BG2S2, A3G3S2, and A2G2S1 glycans (p < 0.05), and FA3G3S3 and A2BG2S2 (p = 0.1). On the contrary, the tetrasialylated glycan FA4G4S4 exhibited a higher abundance in HeLa cells (p = 0.06).

**Figure 6:**
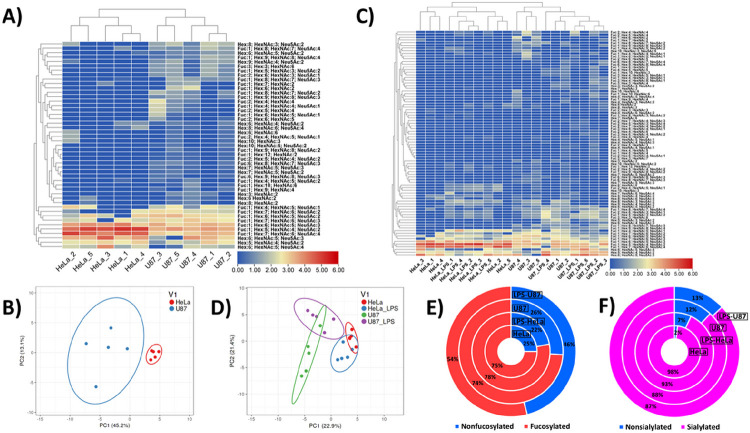
Differential qualitative and quantitative N-glycan profiling of single HeLa and single U87 mammalian cells. **A-C)** Euclidean distance-based hierarchical clustering of N-glycans detected in A) HeLa and U87 cells, and B) LPS-treated and untreated HeLa and U87 cells, based on five repetitive analyses of single HeLa and single U87 cells (labeled from 1 to 5). Red, yellow, and light blue colors correspond to high, medium, and low relative abundances based on the normalized N-glycan signal intensities. N-glycans that are not detected in the cell samples are highlighted in dark blue. **B-D)**Principal component analysis (PCA) of N-glycans detected in HeLa and U87 cells (B), and PCA of LPS-treated and untreated HeLa and U87 cells (D). **E-F)** Total abundances of fucosylated and nonfucosylated (E) and sialylated and nonsialylated (F) N-glycans detected in HeLa and U87 single cells, before and after LPS treatment, based on the normalized N-glycan signal intensities.

Figure 6A depicts the results of non-supervised Euclidean distance-based hierarchical clustering of quantitative glycomic profiles of 47 representative N-glycans detected in HeLa and U87 single cells (i.e., glycans that were detected in at least three CE-MS analyses out of the ten total repetitive analyses). This clustering analysis yielded a reasonable differentiation of HeLa and U87 cell lines, according to their respective single-cell N-glycome profiles. As shown in Fig. 6A and **Table S4A**, HeLa and U87 single cells were clustered into two distinct clades, based on five repetitive analyses acquired for each single-cell type. Glycans like FA2G2S2, FA3G3S3, and FA4G4S4 were clustered into the same clade due to relatively high intensity levels in both HeLa and U87 cell types. Other clades reflected similar intensity levels of relatively medium abundance glycans, e.g., FA3G3S2 and FA4G4S3, in the two analyzed cell types. Interestingly, Man3 and the fucosylated analogs of Man6 and Man12 were only detected in single U87 cells and not in single HeLa cells, while Man6, Man8, and FMan7 were detected in both cell types at similar intensity levels but with a higher frequency in single U87 cells. The previously reported studies on glycomic analysis of U87 or other cells focused on specific proteins’ glycosylation and its biological significance did not provide information on cell surface glycans’ abundances ^57–59^. In contrast, our results present an inventory of single HeLa and single U87 cell surface glycans with their respective glycan compositions and abundances, which allows us to highlight significant and potentially biologically-relevant glycosylation differences between the two cell lines. Principal component analysis (PCA) was also conducted to visualize the dominant trends and underlying specific patterns in the datasets generated by HeLa and U87 single-cell analyses (Fig. 6B). Selecting the 47 representative N-glycans detected in HeLa and U87 single cells above-described (Fig. 6A), two distinct clusters could be clearly differentiated with PCA, corresponding to HeLa and U87 cell lines, respectively (Fig. 6B). The PCA algorithm, therefore, allowed us to confirm the uniqueness and specificity of HeLa and U87 single-cell N-glycomes.

Finally, our proof-of-concept CE-MS^2^ experiments were performed with ~ ten U87 cells and resulted in accurate and unambiguous structural characterization of 29 N-glycans, including sialylated and neutral N-glycans (**Table S5**). Figure 5D shows a characteristic MS^2^ spectrum of **A3G3S5**, detected at a relatively medium abundance in U87 cells. The MS^2^-based structural characterization of this pentasialylated glycan was based on the [M-3H]^3−^ precursor ion at *m/z* 1,152.73. For this glycan, the detection of the C_4α_/Y_5α_^2−^ fragment ion at *m/z* 745.25 and the absence of C_4β_/Y_5β_^1−^ ion at *m/z* 835.28 allowed the assignment of a branched 3-linked antenna ^40^. The typical mass difference of 60.02 Da between the pair of ions ^2,4^A_7_/Y_6_/Y_6_^3−^ (*m/z* 904.97) and ^0,2^A_7_/Y_6_/Y_6_^3−^ (*m/z* 924.98), and ^2,4^A_7_/Y_6_/Y_6_/Y_6_^2−^ (*m/z* 1,212.41) and ^0,2^A_7_/Y_6_/Y_6_/Y_6_^2−^ (*m/z* 1,242.42) confirmed the absence of a core fucose ^55^. Interestingly, ^0,4^A_2_-CO_2_^1−^ diagnostic ion at *m/z* 306.12 was missing despite the relatively high intensity of B_1_^1−^ ion at *m/z* 290.09 (i.e., compared to the intensity level of B_1_^1−^ ion detected in A3G3S4 fragmentation spectrum (Fig. 5C), in which ^0,4^A_2_-CO_2_^1−^ ion could be detected). These results revealed the presence of α−2,3 SiA linkages in A3G3S5.

#### Single-cell N-glycome alterations induced by LPS stimulation.

Previous studies reported that THP-1 mammalian cells treated with lipopolysaccharide (LPS) exhibited increased ^60^ or decreased ^61^ levels of sialylation. Downregulation of glycan fucosylation was also reported for LPS-stimulated brain cells ^62^. To check if the developed CE-MS-based workflow could detect glycome alterations at the single-cell level, HeLa and U87 cells were stimulated with LPS. Interestingly, N-glycan profiling of single HeLa cells, after stimulation of HeLa cells with LPS, resulted in an ~ 3-fold increased number of detected N-glycans, compared to the untreated HeLa cells (Fig. 4J). On average, 37 ± 12 N-glycans per single HeLa cell (n = 5) were identified after LPS treatment, and 76 non-redundant N-glycan compositions were identified in total (Fig. 4K). As shown in Fig. 4E, mono-, di-, and trifucosylated glycans were detected in single HeLa cells following LPS treatment, while N-glycan profiling of single HeLa cells before LPS treatment resulted only in the assignment of mono- and difucosylated glycans. LPS activation of HeLa cells also resulted in the detection of penta- and hexasialylated glycans, undetectable in single HeLa cells before LPS treatment (Fig. 4F). Overall, the fractional distributions of N-glycans detected in single HeLa cells were significantly different before and after LPS treatment. Mono-, di-, and trifucosylated N-glycans accounted for 39%, 13%, and 4%, respectively, in LPS-treated HeLa cells (vs. 51%, 3%, and 0%, respectively, in untreated HeLa cells, Fig. 4E). Mono-, di-, tri-, tetra-, penta-, and hexasialylated N-glycans accounted for 13%, 36%, 11%, 10%, 2%, and 1%, respectively, in LPS-treated HeLa cells (vs. 4%, 34%, 23%, 22%, 0%, and 0%, respectively, in untreated HeLa cells, Fig. 4F). As shown in **Figure S5A**, 34% of the N-glycans detected in LPS-treated HeLa cells were also identified in untreated HeLa cells. Interestingly, 50 N-glycans were uniquely detected in LPS-treated HeLa cells, among which 44% were neutral (including trifucosylated) glycans, and 56% were sialylated (including penta- and hexasialylated) glycans. A quantitative comparison of glycan abundances was also conducted. As shown in Figs. 6E and S5C, the total abundances of fucosylated glycans detected in single HeLa cells were not significantly different (p = 0.6, **Table S3CD**) before and after LPS treatment, and accounted for 75% and 78%, respectively, based on peak intensity measurements. However, the stimulation of HeLa cells with LPS resulted in significantly altered sialylation profiles. As shown in Fig. 6F, a high increase in the total abundance of HeLa cell-derived nonsialylated glycans was noticed (p = 0.06, **Table S3FG**) after LPS treatment. In addition, the total abundances of mono-, tri-, and tetrasialylated glycans were significantly different (p < 0.05, **Table S3FG**) in untreated and LPS-treated HeLa cells (**Figure S5D**). These results confirmed that LPS stimulation of HeLa cells induced significant changes in HeLa cell N-glycome profiles, which could be detected at the single-cell level using our proof-of-concept workflow.

Significant alterations of U87 cell N-glycome profiles were also observed at the single-cell level when U87 cells were treated with LPS, compared to the untreated U87 cells. CE-MS analysis of single U87 cells after LPS treatment resulted in the detection of 55 ± 30 N-glycans per single U87 cell (n = 5), and in the assignment of 161 non-redundant N-glycan compositions in total. 68% of the N-glycans identified in LPS-treated U87 cells were fucosylated, and the fractional distributions were 36%, 15%, 11%, 1%, 1%, and 4% for mono-, di-, tri-, tetra-, penta-, and hexafucosylated N-glycans, respectively (Fig. 4G). Mono-, di-, tri-, tetra-, penta-, hexa-, and heavily (i.e., ≥ 7 SiA residues) sialylated N-glycans accounted for 12%, 22%, 18%, 13%, 2%, 0%, and 1%, respectively (Fig. 4H). As shown in **Figure S5B**, 92 glycans were commonly detected in LPS-treated and untreated U87 cells. Yet, 69 glycans were uniquely detected in LPS-treated U87 cells, among which many pentasialylated and several heavily sialylated N-glycans containing up to 13 SiA residues. These results seem to indicate that LPS treatment induced a modification in the type and structure of biosynthesized sialylated glycans in U87 cells. We think that these unique highly/heavily sialylated glycans, detected on the surface of LPS-stimulated U87 cells at the single-cell level using our developed method, might be challenging to detect using alternative total cellular glycomic analysis or lectin-based methodologies. Indeed, based on our results, the fractional distributions (Fig. 4GH) and fractional abundances (Figs. 6F and S5F**)** of sialylated N-glycans identified in single U87 cells did not change significantly before and after LPS treatment (**Table S3FH**), and only a thorough qualitative and quantitative comparison of released single-cell surface N-glycans could reveal such sialylation subtlety. In addition, the comparative quantitative analysis of fucosylated glycans detected in single U87 cells showed that their total abundances significantly decreased from 74–54% (p < 0.05, **Table S3CE**) when U87 cells were treated with LPS, based on peak intensity measurements (Figs. 6E and S5E). These results clearly demonstrated that LPS treatment induced a downregulation of fucosylated glycans in U87 cells that could be detected at the single-cell level.

Figure 6C and **Table S4B** depict the results of Euclidean distance-based hierarchical clustering of quantitative glycomic profiles of 84 representative N-glycans detected in LPS-treated and untreated HeLa and U87 single cells (i.e., the set of 47 glycans highly representative of HeLa and U87 cells, described above, was extended with 37 representative glycans detected in LPS-treated HeLa and LPS-treated U87 cells, respectively, see Methods section). This clustering analysis resulted in a plausible differentiation of two clades, corresponding to treated/untreated HeLa cells, and treated/untreated U87 cells, respectively. In addition, four repetitive analyses of LPS-treated HeLa cells and four repetitive analyses of LPS-treated U87 cells were clustered together. These results further demonstrated the uniqueness of HeLa and U87 cell line N-glycomes, and showed that both cell lines exhibited different biological responses to LPS treatment. PCA was also conducted on the datasets generated from the analyses of LPS-treated and untreated HeLa and U87 cells, selecting 57 representative glycans (see Methods section) (Fig. 6D). As shown in Fig. 6D, four distinct clusters corresponding to LPS-treated and untreated HeLa cells, and LPS-treated and untreated U87 cells, respectively, were differentiated and confirmed a pronounced effect of LPS treatment. As expected, a partial overlap was observed between the two single HeLa cell-related clusters and the two single U87 cell-related clusters. Overall, these results indicated the undeniable biological effect of LPS stimulation on HeLa and U87 cell N-glycomes, which could be detected at the single-cell level using our developed SCG workflow.

## Discussion

Single-cell omics, spatial omics, and multi-omics are emerging fields in life science, medicine, and fundamental biology applications. Deciphering cell-to-cell variations can be crucial in designing advanced approaches for the diagnosis and treatment of human pathologies. Given the relevance of cell glycosylation analysis for the understanding of tumor initiation, progression, and metastasis, and for identifying effective therapeutic targets, there is a crucial need for the development of SCG methods. In this work, we developed an in-capillary sample processing method for straightforward, unbiased, accurate, and deep qualitative and quantitative N-glycan profiling of single mammalian cells with label-free high-sensitivity CE-MS. Native N-glycans were enzymatically released from the cell surface prior to their CE-MS analysis in the described set of proof-of-concept experiments. To the best of our knowledge, analytical technologies enabling direct and unbiased profiling of single-cell N-glycome have not been reported yet. To date, only a few analytical technologies, based on carbohydrate-binding lectins, have been developed for SCG. These technologies, which require sophisticated instrumentation, are tedious, expensive, and time-consuming and result in undirect and biased profiling of cell surface glycans (i.e., the glycans are not enzymatically released from the cell surface for their direct detection and quantification, and the glycan detectability is highly dependent on lectin binding affinity). Furthermore, every single glycan structure does not have a corresponding lectin and many lectins exhibit low binding specificity toward individual oligosaccharides ^63^. In its current proof-of-concept implementation, the SCG method we developed requires modest but reasonable time per analysis (~ 1 h for the CE-MS analysis and ~ 3 h in total, including cell loading, in-capillary glycan release, and capillary rinses and conditioning), allows a well-controlled injection and processing of individual cells, and requires affordable analytical instruments, compared to the methods reported for total cellular glycomics and lectin-based SCG. Our results showed that the developed CE-MS-based workflow could result in the detection, identification, and quantitation of over 62 N-glycans in one single mammalian cell using the MS equipment we currently have access to.

Specific N-glycosylation patterns were demonstrated for HeLa and U87 single cells, based on a thorough differential analysis of qualitative and quantitative single-cell N-glycome profiles. A substantially higher number of N-glycans (~ 5-fold) was detected on the surface of single U87 cells, compared to single HeLa cells, which may be attributed in part to the larger size of U87 cells. In addition, significant differences in the fractional distributions and abundances of the N-glycans detected in HeLa and U87 single cells were observed, reflecting unique molecular features for each cell type. Interestingly, N-glycome alterations were observed at the single cell level when HeLa and U87 cells were stimulated with LPS, which manifested the change in the phenotypic cell state reflected on the cell surface. Notably, a significantly higher (~ 3-fold) number of N-glycans and higher levels of fucosylation and sialylation were detected in LPS-treated HeLa cells, compared to untreated HeLa cells. On the other hand, the stimulation of U87 cells with LPS induced the downregulation of fucosylated glycans, compared to the untreated U87 cells. Overall, we demonstrated in the presented here proof-of-concept study that our developed SCG workflow could effectively and accurately characterize the single-cell N-glycome of different mammalian cell lines and detect N-glycome alterations at the single-cell level. The acquired results demonstrated the potential of the technique to differentiate cell phenotypes, states, types, and lineages based on alterations of N-glycan representation on the cell surface. The developed approach is mild, which allowed us to preserve the cell integrity during the enzymatic release of glycans, which may potentially benefit the multi-omic characterization of individual cells.

CE-MS analysis of N-glycans in their native non-labeled state enabled the preservation of glycans’ integrity and endogenous structural features, especially fucosylation and sialylation. Highly fucosylated (up to 6 fucose residues) and heavily sialylated (up to 13 Neu5Ac residues) N-glycans were detected in single U87 cells. Highly fucosylated and heavily sialylated N-glycans were also detected in HeLa cells but in larger injected amounts of HeLa cells (5–10 cells), indicating the extremely low abundances of such glycans in HeLa cells. CE-MS^2^ analyses performed in negative ESI mode resulted in unambiguous and accurate characterization of > 80 N-glycan structures in the analyzed mammalian cells and enabled unequivocal identification of core fucose or outer-arm fucose residues and antennary branching. Besides, the presence of α−2,6 Neu5Ac linkages could also be detected in many glycans, based on the detection of the characteristic diagnostic ion ^0,4^A_2_-CO_2_^1−^. CE-MS^2^ analyses also resulted in the structural characterization of neutral N-glycans, including high mannose-type N-glycans.

In this study, we also demonstrated the potential of the newly developed workflow for deep and highly informative N-glycan profiling of tiny amounts of human blood-derived isolates, including extracellular vesicles, which are important intracellular communicators with diagnostic potential. Over 132 and 88 N-glycans were detected in IgM and IgG for injected amounts of 100 pg (i.e., 100 amol) of IgM, and 500 pg (i.e., 3 fmol) of IgG, corresponding to only ~ 10 pg of N-glycans and ~ 50–60 pL of human serum. These minute amounts of proteins and glycans are estimated to be equivalent to the protein and glycan content of one single mammalian cell. CE-MS analysis of total plasma resulted in the identification of > 234 and > 152 N-glycans for injected amounts of approximately 50 pL and 5 pL of human plasma, respectively (i.e., ~ 150 and ~ 15 pL of blood, respectively). Over 226 and 127 N-glycans were detected in total EV isolate, using injected amounts equivalent to ~ 150 nL and ~ 3 nL of plasma, respectively. The numbers of N-glycans identified in IgM, IgG, total plasma, and total EV isolate reported here, using sub-0.5 ng-levels of serum proteins and nL/pL-levels of plasma isolates, largely exceed (~ 7-fold) those reported in other N-glycan profiling studies of similar complexity blood-derived isolates ^40,41,47–50,53^. These results further demonstrate the impressively high sensitivity of the newly developed glycan profiling method, which allowed us to increase the depth of glycan profiling (i.e., detect higher numbers and varieties of glycans) and, therefore, expand the glycan catalog of the four types of analyzed blood-derived isolates.

We envision that our approach can open new doors in the field of glycomic profiling of scarce samples and single-cell glycomic research, and it can be extended to the analysis of a large variety of glycans (e.g., O-glycans and lipoglycans) as well as to the glycan profiling of other biological and clinically-relevant amount-limited samples. We expect that the developed workflow will provide a wealth of information on eukaryotic (or prokaryotic) cell and EV glycomes and will enable differential glycomic profiling studies of cell and EV subpopulations. The developed method is a promising approach for identifying new glycan biomarkers in human pathologies using limited amounts of biological materials, e.g., liquid microbiopsies, small populations of cells or EVs, and single cells (and even single microvesicles or small populations of microvesicles). The innovative SCG technique could also be potentially integrated with other omic approaches in a single-cell multi-omics platform. Multi-omic and spatial profiling of individual cells could provide crucial information on biological mechanisms underlying complex diseases, which is unachievable by merging data sets obtained from mono-omics studies of different cells and bulk samples.

Over the past decades, chemical tools have been developed to profile cell surface glycans and get insights into their spatial distribution and dynamic turnover for a better understanding of their specific role in the development and progression of human diseases ^64^. For example, the Bertozzi group pioneered chemical reporter strategies, including metabolic labeling of cell surface glycans with azido groups, for visualizing and monitoring glycan dynamic changes in living cellular organisms ^63,65^. We think that our developed SCG approach could serve as an alternative and complementary technique for a quick, effective, and sensitive analysis of cell surface glycans in small populations of cells and single cells. Our CE-MS-based label-free strategy, which has the capability to detect a large variety of glycans (including peculiar glycans such as heavily sialylated glycans) in their native state, could help monitor cell surface glycosylation changes that regulate cellular functions during cell growth, differentiation, activation, proliferation, and survival. In the relatively nascent and highly challenging field of SCG, an arsenal of cutting-edge technologies has to be developed and combined in joint efforts for routine screening of single-cell glycomes. We hope that our proof-of-concept study will help shed light on the complexity of cell surface glycans and their roles in the biology of the cell in health and disease.

## Data Availability

All data required to evaluate the results and conclusion discussed in the manuscript are present in the main manuscript and in the Supplementary Information. The raw data generated in this study have been deposited in GlycoPOST (GPST000378) (https://glycopost.glycosmos.org).
